# Selenium Speciation in Se-Enriched Soybean Grains from Biofortified Plants Grown under Different Methods of Selenium Application

**DOI:** 10.3390/foods12061214

**Published:** 2023-03-13

**Authors:** Maila Adriely Silva, Gustavo Ferreira de Sousa, Gary Bañuelos, Douglas Amaral, Patrick H. Brown, Luiz Roberto Guimarães Guilherme

**Affiliations:** 1 Department of Soil Science, Federal University of Lavras, Lavras 37200-900, MG, Brazil; 2 USDA Agricultural Research Service, San Joaquin Valley Agricultural Sciences Center, 9611 S. Riverbend Avenue, Parlier, CA 93648-9757, USA; 3 Agriculture and Natural Resources, University of California, 680 Campus Drive, Hanford, CA 93230, USA; 4 Department of Plant Science, University of California, One Shields Ave, Davis, CA 95616, USA

**Keywords:** selenium amino acids, biofortification, selenium fertilizers, food composition, selenomethionine, food security

## Abstract

Since soybean is widely cultivated around the world and has a high protein content, it is a great nutritional vehicle for increasing the dietary uptake of selenium (Se). Several studies have evaluated biofortification with Se through fertilizer application in several crops. However, it is not clear how each method and source affect the total Se content or Se species in soybean grains. This work aimed to assess the total Se content and Se speciation in Se-enriched soybean grains produced under different Se application methods in the field. The treatments consisted of Se application (soil or foliar), using organic or inorganic Se sources at 10 g ha^−1^ or 80 g ha^−1^, in two genotypes. The results showed that all treatments with inorganic Se (soil and foliar) increased the Se content in grains compared with the control. More than 80% of the total Se in grains was present as selenomethionine (SeMet), and the speciation was affected by the Se source and the method of application. The treatments using inorganic Se, applied via soil or foliar, produced the highest content of Se as SeMet in soybean grains. Finally, we propose that the preservation of the Se species in products derived from soybean grains be evaluated as the following step.

## 1. Introduction

Selenium (Se) is a micronutrient for animals and humans and is needed for hormone regulation, antioxidant defenses, and immune and muscle systems [1]. The average intake recommendation for adults is 55 μg day^−1^, yet it can reach 60 and 70 μg day^−1^ in pregnant and lactating women, respectively [2]. Up to one in seven people worldwide are estimated to have deficient dietary Se intake [3]. The reduced Se intake occurs mainly because agricultural technologies in many parts of the world have focused on promoting grain yield, rather than on increasing the nutrient content [4].

Soil Se is the primary natural source of Se in staple crops, but its availability is largely affected by parent rocks, climate, and soil chemistry (e.g., soil pH and redox potential) [5,6]. Moreover, the Se soil content is not always enough to supply the daily-recommended intake of 55 μg day^−1^ for the population. Disorders related to Se deficiency are more common in regions with severely low Se content in soils [2,7,8], including soils in the tropics [6].

Selenium can be added to the diet of humans by supplementing foods directly or by biofortifying plants, i.e., by increasing Se levels in crops during plant growth/development. Biofortification can be performed through conventional plant breeding or modern biotechnology (genetic biofortification) or by increasing Se uptake via Se supplementation to plants (agronomic biofortification) [4,9,10,11]. Agronomic biofortification has been effective in increasing the content of Se in several grain crops, such as wheat [12,13], rice [14], common bean [15,16], and sorghum [17].

The effectiveness of Se fertilization in grain crops is highly affected by genotype, chemical source applied, and method of Se application [18,19]. The main strategies for agronomic biofortification are through soil and/or foliar applications, though some authors have suggested that foliar supply is more efficient for increasing the Se content than soil application supply due to the direct contact of the element with the crop and prevention of Se sorption in the soil [20,21].

Both the total content and the bioavailability of Se in food exert a great role in Se assimilation by humans and animals. Selenium species are frequently found in plants in both organic Se compounds (i.e., selenomethionine (SeMet), methyl selenocysteine (MeSeCys), selenocysteine (SeCys)) and inorganic compounds (i.e., selenite (SeO_3_^2−^), selenate (SeO_4_^2−^)) [22]. Selenium bioavailability differs based on the chemical form of the element present in the food and is considerably greater for organic forms [23].

The variation in characteristics of different genotypes has a significant influence on Se content and on its chemical forms in edible parts [24,25]. Indeed, Se biofortification efforts may profit from this natural variation among plant genotypes, choosing those that can naturally accumulate higher Se levels in edible parts. In this context, soybean has been suggested to be a promising alternative to producing biofortified cereal grains given its higher protein content and, hence, greater propensity to produce organic Se forms such as SeMet, MeSeCys, and SeCys [26]. Soybean is one of the most produced grains in the world, which makes this crop a good Se vehicle for increasing Se in the population. This study aims to evaluate the Se content and speciation in Se-enriched soybean grains produced under different Se application methods in the field.

## 2. Materials and Methods

### 2.1. Area Characterization

Soybean target grain samples were selected from three different trials previously grown under the same field conditions [27], during the season 2018/2019 in Capão Bonito (SP), Brazil (Lat: −95 24.040934, Lon: −48.262421). The target samples were selected for the study based on earlier analyses of the total Se content in Se-enriched grains under different Se application conditions. The average annual precipitation in the cultivated field was 1628 mm, and the average annual temperature was 18.8 °C. The soil of the experimental region is an Oxisol, classified as Typic Hapludox [28]. Physical and chemical characteristics were determined according to the methodology suggested by the Brazilian Agricultural Research Company [29] as follows: pH (H_2_O) = 6.0; H + Al = 2.96; Al = 0.06; P (Mehlich-1) = 34.8 mg dm^−3^; P-rem = 28.10 mg L^−1^; K = 148 mg dm^−3^; S = 4.11 mg L^−1^; CEC = 9.83 cmol_c_ dm^−3^; Ca = 5.05 cmol_c_ dm^−3^; Mg = 1.44 cmol_c_ dm^−3^; organic matter = 2.69 dag dm^−3^; Se = 0.92 mg dm^−3^; clay = 510 g kg^−1^; silt = 110 g kg^−1^; and sand = 380 g kg^−1^.

### 2.2. Selenium Application Methods

The treatments comprised both soil and foliar application of Se (Table 1). When Se was applied to soil, two different phosphate fertilizers coated with Se were used at planting time (conventional monoammonium phosphate and enhanced efficiency monoammonium phosphate). The fertilizers were coated with 500 mg kg^−1^ of Se after granulation (using a solution of sodium selenate—Na_2_SeO_4_, Sigma-Aldrich, Saint Louis, MO, USA). Given that 80 kg ha^−1^ of P_2_O_5_ was applied as MAP (~50% P_2_O_5_), the addition of Se-rich fertilizers (500 mg Se kg^−1^) resulted in a Se dose of 80 g ha^−1^.

Foliar spray with an inorganic Se source was applied at two doses, 10 and 80 g ha^−1^, as sodium selenate (Na_2_SeO_4_—Sigma Aldrich 98.9%, Saint Louis, MO, USA). Another treatment with an organic Se source (acetylselenide—25% of Se) was applied at 10 g ha^−1^ via foliar spray. Fertilizers were applied at phenological stages R3 (beginning of pod development) and R5 (grain filling), with each application containing half of the total rate. Sodium selenate was diluted in deionized water (500 mL of water plot^−1^) before being applied with a pressurized carbon dioxide pump, and no Se application was used as a control treatment for both genotypes. Mineral oil was used with each application solution (0.5% *v*/*v* of mineral oil). All treatments were applied to two different genotypes (50I60 Lança and M5917) (Table 1).

### 2.3. Selenium Extraction: Soluble and Protease

The samples from each treatment were dried at 40 °C (to avoid protein denaturation) in a drying oven with air circulation after being harvested. In addition, they were ground in an electric hand mill (ka-A11 basic BS32, IKA, Staufen, Germany). Analyses of total Se and Se speciation in the Se-enriched soybean grain followed the methodology described by Bañuelos et al. (2012) and Bañuelos et al. (2012) [22,30]. Soluble Se compounds (non-protein bound) and insoluble Se compounds (protein-bound) were separated and identified/quantified using methanol-chloroform-water solvent extraction and methanol-chloroform-water enzymatic digest (with protease), respectively.

The methanol-chloroform-water extraction was performed using 1 g of the ground sample placed in 40 mL glass vials equipped with Teflon caps and divided into two groups (soluble and protease). Protease (*Streptomyces griseus* Type XIV—Sigma-Aldrich) was added (50 mg) to the protease set [31]. Following that, 10 mL of ultrapure water at room temperature was added to the protease-containing vials. The set of soluble samples received 17 mL of methanol. The matched samples were mixed by vortex, and the protease sample set was maintained at 37 °C for 20 h, while the methanol sample set was kept at 4 °C overnight. Protease-digested samples received 17 mL of methanol, and soluble-digested methanol extractions received 10 mL of water (to inhibit enzymatic action and denature the protease enzyme).

Each tube was stirred continuously and left in a refrigerator at 4 °C overnight. Then, chloroform (8.5 mL) was joined to all vials, which were sealed, shaken quickly, and refrigerated at 4 °C overnight until the material was totally extracted. The top aqueous phase (methanol-water) was completely separated from the chloroform phase. This phase (methanol-water) containing the extracted Se compounds was removed and put into a centrifuge tube. One-quarter of the aqueous phase (methanol-water) was then pipetted into 50 mL ICP digestion tubes for drying (using a heating block at 50 °C), which was followed by acid digestion and analysis of total aqueous Se by Agilent 7500 CX ICP-MS (Agilent Technologies, Santa Clara, CA, USA). Additionally, the nonaqueous chloroform phase remaining in the original 40 mL glass vial was also evaporated, then acid-digested and analyzed with ICP-MS. After extraction, the portion of Se in the aqueous phase was calculated as {(total Se in methanol-water phase)/[(total Se in methanol-water phase) + (total Se in chloroform phase)] × 100}.

The final part of the aqueous (methanol-water) phase was dried using a refrigerated centrifugal speed vacuum (Labconco CentriVap Concentrator), resuspended in ultrapure water to 2.5 mL, and then saved in a −80 °C freezer. Waters Se-Pak Classic C18 cartridges (360 mg 55+ 05 um) were utilized for the full cleanup of the aqueous concentrates. Each cartridge was cleaned by flushing it with 10 mL of methanol and 5 mL of ultrapure water in that order. The 2.5 mL concentrates were thawed and vortexed, and then received 11 μL of formic acid (88%—ACS grade, Fisher Chemical) before being transferred to the Sep-Pak. The sample was eluted using a syringe into a new 50 mL conical tube. Methanol (3 mL) was added to remove residuals in the conical tube. Afterward, the aliquot was dried, dissolved in 1.5 mL of water, placed in Agilent screw-top glass HPLC vials, and frozen (−80 °C) until SAX-HPLC/ICPMS analysis.

The National Institute of Standards and Technology (NIST) wheat flour standard (SRM 1567a) was used as the standardized quality control for wet-acid digestion (total Se concentration) and Se speciation extraction (SeMet, SeCys2) content in plant material. The SRM 1567a was utilized as an internal control in the MCW extraction to account for any changes in the protease XIV efficacy and other factors during the extraction process. The total Se recovery rates were over 94% for the wheat flour standard, which has a Se concentration of 1.1 ± 0.2 μg Se g^−1^ DW, with a method detection limit of 50 ng Se g^−1^ DW. The selenoamino acid content in SRM 1567a consisted of 92% SeMet and 6% SeCys2. The NIST wheat flour standard and soybean samples were extracted in triplicate. The extraction and quality control measures are documented in detail [30].

### 2.4. Total Selenium, Selenium Recovery, Selenium Speciation, and Total Free Amino Acids

Total Se concentrations were analyzed by Agilent 7500 CX ICP-MS (Agilent Technologies Santa Clara, USA) [22,30]. The fraction of applied Se incorporated in soybean grains (Se recovery) was calculated using Se total data, as follows:

Se recovery (%) = (Se_treatment_ − Se_control_)/Se_dose_ × 100 (1)

where: Se_treatment_ (g ha^−1^) = Se contents in soybean grains from soybean plants grown in treatments that received Se applications; Se_control_ (g ha^−1^) = Se contents in soybean grains from soybean plants grown in treatments without Se applications; Se_dose_ (g ha^−1^) = Se doses applied in the treatment.

Selenium speciation was performed on an Agilent 1200 HPLC connected to a Hamilton PRP-X100 strong anion exchange column (10 μm particle size, 250 mm length, and 4.1 mm internal diameter) coupled to the Agilent 7500 CX ICP-MS (SAX-HPLC-ICP-MS). The Agilent Chemstation software was used to combine the two instruments (Agilent 1200 HPLC and Agilent 7500 CX ICP-MS) with chromatographic data analysis. To account for any matrix-induced changes to the chromatographic analysis, the retention time of Se78 containing peaks was monitored using the ICP-MS and directly compared with the authentic standard [30]. The ICP-MS was operated in collision/reaction cell mode with hydrogen at 5 mL/min to minimize isobaric interferences. The limits of detection (LOD = 3 σ/m) for the primary 5 selenium species speciated ranged from 0.16 to 0.32 ng mL^−1^. The limits of quantification (LOQ = 10 σ/m) for all 5 respective Se species ranged from 0.56 to 1.02 ng mL^−1^. The correlation coefficients ranged from 0.9997 to 0.9999 for all calibration curves. Standards of sodium selenate, sodium selenite, SeMet, and SeCys from Sigma-Aldrich, St. Louis, MO, and methyl-selenocysteine (MeSeCys) from Pharma Se were used for analyses [22,30]. The ninhydrin method was used to assess total free amino acids [32].

### 2.5. Statistical Analyses

Analysis of variance (ANOVA) was performed to verify the effects of treatments on the attributes analyzed. Selenite, Selenate, and SeCys data were log-transformed and fitted to a linear model to address the statistical assumptions before ANOVA (normality and homoscedasticity). Due to homoscedasticity, the SeMet and MeSeCys data were also evaluated using linear models estimated by generalized least square regression. A Tukey test (5% of probability) was performed to compare the treatments. A principal component analysis (PCA) was carried out to report the interaction between Se species. The simple linear relationship between the Se species was performed using Pearson’s correlation. All statistical analyses were carried out using R [33].

## 3. Results

### 3.1. Selenium Accumulation, Recovery, and Speciation in Soybean Grains

**Figure 1:**
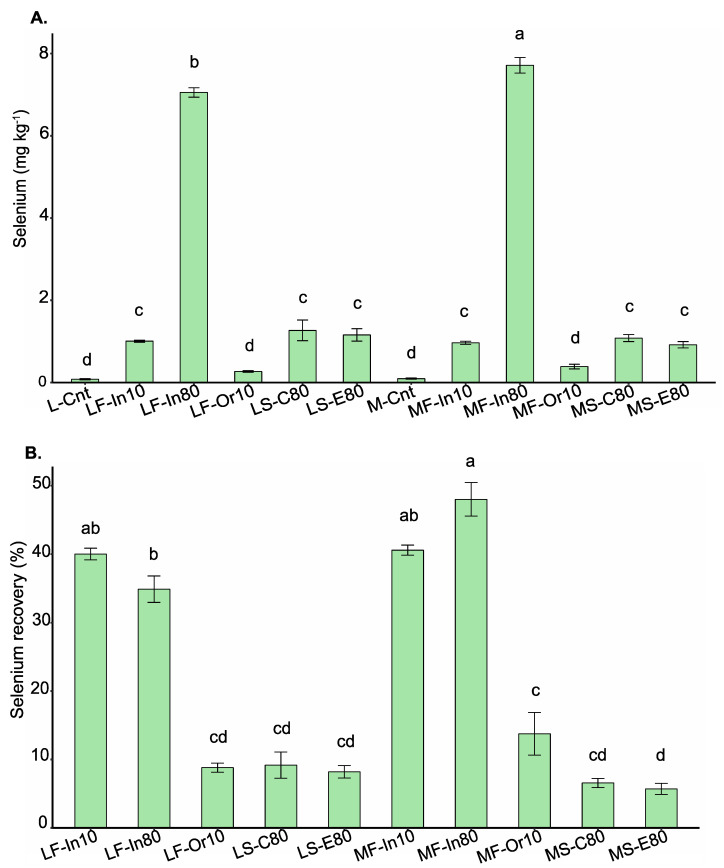
Selenium content (mg kg^−1^) (**A**) and selenium recovery (%) (**B**) in soybean grains from biofortified plants growing under different methods of selenium application. Lowercase letters compare Se contents and Se recovery among the treatments at the level of 5% by the Tukey test. The vertical bars represent the standard error (*n* = 4). Genotype: L = Lança and M = M5917; Se application: F = foliar and S = soil; Se source: In = Se inorganic, Or = Se organic, C = C-MAP + Se, and E = E-MAP + Se; Rate: 10 = 10 g ha^−1^ and 80 = 80 g ha^−1^; Cnt = control.

Total Se concentrations in soybean grains ranged from 0.08 to 7.71 mg kg^−1^ for treatments L-Cnt and MF-In80, respectively (Figure 1A,). All treatments with inorganic Se application (soil and foliar) increased the Se content in grains compared with the control (Tukey 0.05 probability). The MF-In80 treatment produced grains with higher Se content than the LF-Ino80 treatment, indicating that total Se in the genotype M5917 was greater than in the genotype Lança when sodium selenate was applied at a dose of 80 g Se ha^−1^.

Treatments LF-Or10 and MF-Or10, in which Se was applied via an organic source at a dose of 10 g ha^−1^, did not differ from L-Cnt and L-Cnt for total Se analysis. Treatments with Se foliar application using sodium selenate at a dose of 10 g ha^−1^ (LF-In10 and MF-In10) were similar to treatments with Se soil application via phosphate fertilizer at a dose of 80 g ha^−1^ (LS-C80, LS-E80, MS-C80, and MS-E80). The total Se concentrations were 1.27, 1.16, 1.08, and 0.92 for LS-C80, LS-E80, MS-C80, and MS-E80, respectively. There was no significant difference in total Se content between conventional and enhanced efficiency phosphate fertilizer applications.

The treatments MF-In80, MF-In10, LF-In10, and LF-In80 showed higher percentages of Se recovery by grains (48.0%, 40.6%, 40.0%, and 34.9%, respectively) (Figure 1B). The percentage of Se recovery by soybean grains ranged from 5.7% to 48%. The treatment LF-In80 (foliar application) provided a recovery that was 3.8 and 4.3 times greater than LS-C80 and LS-E80 (soil application), respectively. For the genotype M5917, this recovery was 7.3 and 8.4 times greater than the treatments MS-C80 and MS-E80, respectively.

**Figure 2:**
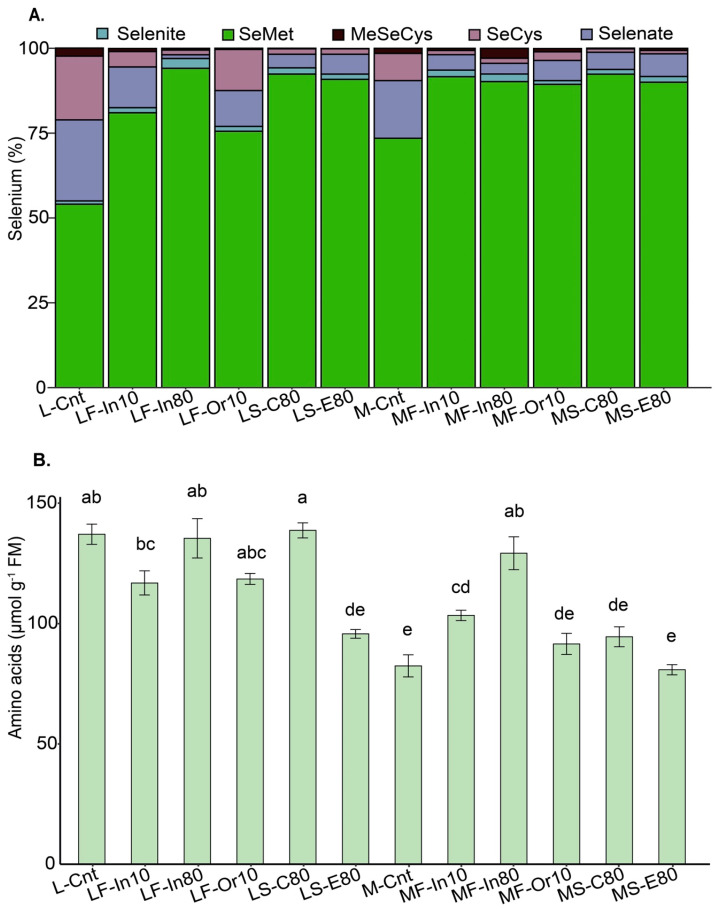
Selenium species (%) (**A**) and amino acids (µmol g^−1^ FM) (**B**) in soybean grains from biofortified plants growing under different methods of selenium application. Lowercase letters compare Se contents and Se recovery among the treatments at the level of 5% by the Tukey test. The vertical bars represent the standard error (*n* = 4). Genotype: L = Lança and M = M5917; Se application: F = foliar and S = soil; Se source: In = Se inorganic, Or = Se organic, C = C-MAP + Se, and E = E-MAP + Se; Rate: 10 = 10 g ha^−1^ and 80 = 80 g ha^−1^; Cnt = control.

The percentages of Se species found in soybeans are shown in Figure 2A. Selenium species in soybeans were influenced by the treatments applied (*p* ≤ 0.05) (Table S1). For SeCys, the percentages ranged from 0.97% (MS-E80) to 17.0% (L-Cnt). The overall percentage of MeSeCys did not differ statistically among the treatments. In general, the Se species with the lowest percentages were MeSeCys and selenite. The highest average percentages for selenite species were found in the LF-In80 treatment (2.3%), yet this treatment differed only from the M-Cnt.

The Se species found in the highest percentage in the grains was SeMet, which ranged from 54.0% (L-Cnt) to 94.1% (LF-In80). The LF-In80 treatment had the highest SeMet mean (94.1%); however, it differed statistically only from the LF-Or10 and M-Cnt. Organic Se (including SeMet, SeMeCys, and SeCys) accounted for more than 84% of the total Se content in the grains following Se applications. In summary, the percentages of Se species present in the grains were higher for SeMet, followed by selenate, SeCys, selenite, and MeSeCys.

### 3.2. Total Free Amino Acids

The total free amino acid concentrations ranged from 80.8 to 138.7 µmol g^−1^ FM, with the LS-C80 treatment showing the highest mean (Figure 2B). The LS-C80 treatment had an average similar to the L-Cnt, LF-In80, LF-Or10, and MF-In80 treatments. The treatments M-Cnt and MS-E80 showed the lowest averages for total free amino acids, according to the results. Comparing the treatments of genotype M5917, the MF-In80 treatment showed the highest concentration of total free amino acids (129.2 µmol g^−1^ FM). In general, treatments of the genotype Lança had a higher content of total free amino acids than the others.

### 3.3. Principal Component Analysis

**Figure 3:**
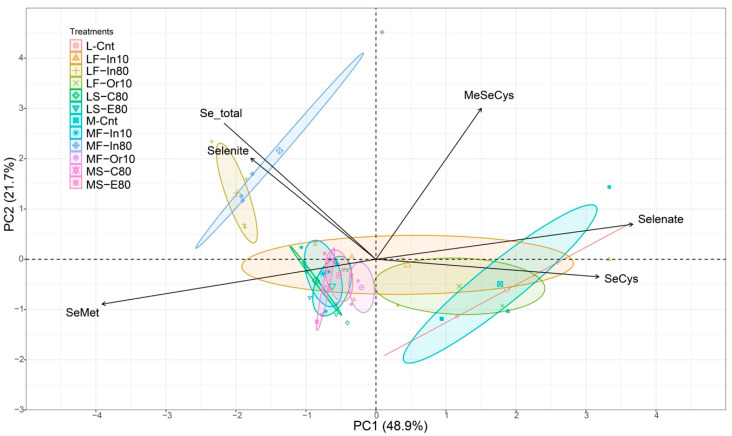
Principal component analysis. Abbreviations: SeMet = selenomethionine; MeSeCys = methyl selenocysteine; SeCys = selenocysteine. Genotype: L = Lança and M = M5917; Se application: F = foliar and S = soil; Se source: In = Se inorganic, Or = Se organic, C = C-MAP + Se, and E = E-MAP + Se; Rate: 10 = 10 g ha^−1^ and 80 = 80 g ha^−1^; Cnt = control.

The principal component analysis (Figure 3) demonstrated that two main components accounted for 70.6% of the variation in Se species. The variable responses influenced by treatments with low Se content in grains (L-Cnt, M-Cnt, and LF-Or10) are shown on the right, indicating an increase in the content of selenate and SeCys in grains. In addition, SeMet content in these treatments was lower and it decreases as selenate content increases. This information was also confirmed by Pearson’s correlation analysis (Table S2). The other group shown on left (up to the horizontal line) is influenced by the treatments with high foliar-applied Se doses, for both genotypes (LF-In80 and MF-In80). This group indicates an increase in the Se total and selenite in grains.

## 4. Discussion

The results demonstrate that soybean is responsive to both soil and foliar biofortification with Se fertilizers, yet the Se concentration in grains depends on how Se is applied. Even though Se is not established as a nutrient for plants, it is clearly a beneficial element [34] as it can enhance plant development and increase antioxidant capacity, especially when plants are exposed to stressing conditions [16,35,36].

All treatments that used inorganic Se—in both foliar and soil application—increased the total Se content in soybean grains. Deng et al. (2021) [37], using pot experiments with soil application of Se, observed that Se content was easily increased in soybean seeds resulting in a 21–27% greater Se content than in the control treatment. Even at a lower rate, treatments LF-In10 and MF-In10 achieved an average total Se content comparable to LS-C80, LS-E80, MS-C80, and MS-E80. In rice crops, Lessa et al. (2020) [14] found that foliar application was more effective in enhancing the quality of Se content in grains than soil application of Se associated with phosphate fertilizers.

In the present study, the Se concentration achieved 7.71 mg kg^−1^ when Se was foliar sprayed in treatment MF-In80. The high recovery of Se in this treatment is due to the higher uptake efficiency of Se associated with a high distribution efficiency of Se from leaves to grains. Similarly, Se foliar application at the highest dose of Se improved the recovery efficiency of Se in the genotype M5917 (∼1.3-fold) when compared with the genotype Lança in the present study. These findings suggest that different genotypes have varying capacities for Se uptake following foliar application with Se inorganic sources, particularly at high doses of Se application.

Foliar spraying transfers Se directly from the leaves to the grains, making it more bioavailable than when Se is applied to the soil [38]. Indeed, when Se is soil applied, it is subjected to various reactions that affect its mobility and solubility [20,39,40]. As a result of the changes in Se mobility and solubility, the efficiency of Se absorption and distribution by plants is also impacted. Soil redox potential, soil texture, sorption/desorption reactions, pH, presence of competing ions, and dissolution processes in soils are the factors that affect Se availability in the soil, affecting the absorption of Se by plants [39,40,41,42].

The bioavailability of Se for animals and humans is based on the Se species consumed and the total Se content [43]. Consequently, the speciation of Se in plant tissue is important for understanding the efficiency of Se absorption. When Se is taken up by the plant, it is transformed into different species in plant tissues. For vegetables, such as garlic, onion, and broccoli, MeSeCys is the main Se species of Se, while in most grains, the main Se species is SeMet [36,44]. Soybean can accumulate Se both as organic and inorganic forms of Se. Here, we found that SeMet was the main Se species produced. These results corroborate the results found by Lu et al. (2018) [45] and Deng et al. (2021) [37]. According to Bañuelos et al. (2020) [46], most non-Se-accumulating plant species are expected to accumulate Se as SeMet.

Plants utilize sulfate transporters to uptake Se when it is provided as selenate [47,48]. The physical and chemical similarity of Se—as selenate- and S—as sulfate—indicates that both elements shared similar metabolic pathways in plants [49]. The transformation of Se into selenite is the first step of assimilation. Later, the cysteine-synthase enzyme helps change selenite into selenide, which is then changed into SeCys. Based on the species and surrounding circumstances, SeCys can then be transformed into elemental Se, MeSeCys, or SeMet. The SeMet species can be either transformed into methyl-SeMet (Me-SeMet) or used to produce selenoproteins [50]. The percentages of Se species found in grains are influenced by how Se is applied. The treatments L-Cnt and M-Cnt accumulate less SeMet and more SeCys than those that received inorganic Se supply via soil application. In other words, under low Se availability, soybeans did not form as much SeMet as when this plant is under high Se availability.

Selenoamino acids, such as SeMet and SeCys, are key for the formation of selenoproteins in plants. The production of SeMet and SeCys in selenoproteins offers even more benefits because they are known to have promising biological properties in vitro and in vivo against free radicals such as HO^•^ and O_2_^−^ [51]. Selenoproteins, such as glutathione peroxidase, play important roles in Se transport and cellular redox balance regulation [52]. Organic Se forms (e.g., Se amino acids) are more bioavailable for humans than inorganic forms such as selenite and selenate. In fact, the human body takes more than 90% of SeMet but only about 50% of Se from selenite [10,53].

Total free amino acids varied according to the treatments applied. The treatment MS-E80 had an amino acids content that was higher than MS-C80, MS-E80, MF-In80, MF-In10, M-Cnt, and MF-Or80. This observation indicates that foliar application of Se at a dose of 80 g ha^−1^ increased the total free amino acids for genotype M5917. However, this result was not observed for treatments applied in genotype Lança, which showed a variable response among the treatments. In lettuce accessions, Ramos et al. (2011) [54] reported a large discrepancy in total free amino acids using selenate and selenite as Se sources. In contrast, Huang et al. (2022) [55] found an increase in total amino acids in soybean sprouts that received Se at concentrations smaller than 40 mg L^−1^ and a decrease in sprouts treated with Se greater than 60 mg L^−1^. According to these authors, low-level Se doses promote amino acid synthesis, whereas high-level Se doses have an inhibitory effect. In general, the treatments with high total free amino acids were L-Cnt, LF-In80, LS-C80, and MF-In80. According to Sanmartín et al. (2014) [56], adequate Se accumulation in plants can enhance their nutritional values by increasing the total amino acid content.

## 5. Conclusions

Our results show that different methods of Se application in soybean plants affect total Se and Se species in soybean grains. Foliar application using inorganic Se at 80 g ha^−1^ is more efficient for improving total Se content in soybean grains, for both genotypes. The foliar spray at the dose of 10 g ha^−1^ provides Se content in the grains similar to that found as a result of the application of 80 g ha^−1^ via soil. More than 80% of total Se in soybean grains was present in the organic form, with SeMet as the main Se species accumulated by soybean grains. The highest levels of SeMet in soybean grains were obtained in the treatments with the application of inorganic Se, either via soil or via foliar application, in both genotypes evaluated. In order to assess the mechanism by which Se sources and Se carriers (i.e., the different soil fertilizers) affect Se species in soybean grains, further research is required. Lastly, we propose future trials to evaluate the preservation of the Se content and Se species in products originating from soybean grains after industrial processing.

## Figures and Tables

**Table 1:** Treatments description applied in soybean plants.

Abbreviation	Genotype	Method of Application	Se Source/Vehicle	Dose (g ha^−1^)
L-Cnt	Lança	-	-	0
LF-In10	Lança	Foliar	Inorganic ^1^	10
LF-In80	Lança	Foliar	Inorganic	80
LF-Or10	Lança	Foliar	Organic ^2^	10
LS-C80	Lança	Soil	C-MAP ^3^ + Se	80
LS-E80	Lança	Soil	E-MAP ^4^ + Se	80
M-Cnt	M5817	-	-	0
MF-In10	M5817	Foliar	Inorganic	10
MF-In80	M5817	Foliar	Inorganic	80
MF-Or10	M5817	Foliar	Organic	10
MS-C80	M5817	Soil	C-MAP + Se	80
MS-E80	M5817	Soil	E-MAP + Se	80

^1^ Inorganic Se source = sodium selenate; ^2^ organic Se source = acetylselenide; ^3^ phosphate fertilizer applied via soil = C-MAP: conventional monoammonium phosphate; ^4^ phosphate fertilizer applied via soil = E-MAP: enhanced efficiency monoammonium phosphate (coated with humic and fulvic substances).

## Data Availability

The data presented in this study are available from the corresponding author upon reasonable request.

## Supplementary Materials

The following supporting information can be downloaded at: https://www.mdpi.com/article/10.3390/foods12061214/s1, Table S1: Means (% of Se species) and Tukey test for Se species. Table S2: Correlation coefficients among Se species in soybean grains.
