# Supplementary material for: Selenium Speciation in Se-Enriched Soybean Grains from Biofortified Plants Grown under Different Methods of Selenium Application
Source: Foods. 2023 Mar 13;12(6):1214. doi: 10.3390/foods12061214 (PMC10048027; doi:10.3390/foods12061214)
Supplement: Supplementary file 1 [file foods-12-01214-s001.zip › foods-2212022-supplementary.pdf]

Supplementary Material

Table S1: Means (% of Se species) and Tukey test for Se species.

Abbreviation	SeMet	SeCys	MetSeCys	Selenite	Selenate
L-Cnt	54.00 abc	17.00 a	2.38 a	0.87 a	23.88 ab
LF-In10	81.00 abc	4.09 bc	0.90 a	1.23 a	12.00 ab
LF-In80	94.10 a	1.29 d	0.53 a	2.34 a	1.10 b
LF-Or10	75.50 bc	11.25 ab	0.35 a	0.89 a	10.57 a
LS-C80	92.40 a	1.49 cd	0.18 a	1.24 a	4.00 b
LS-E80	90.80 a	1.51 cd	0.10 a	1.10 a	5.95 ab
M-Cnt	73.50 c	7.86 ab	1.50 a	0.00 b	16.98 ab
MF-In10	91.60 a	1.24 d	0.63 a	1.52 a	4.55 ab
MF-In80	90.20 a	1.20 d	2.93 a	2.18 a	3.15 b
MF-Or10	89.40 ab	2.38 cd	0.95 a	1.00 a	5.95 ab
MS-C80	92.4 a	1.02 d	0.20 a	0.65 ab	5.05 ab
MS-E80	90.0 a	0.97 d	0.63 a	1.37 a	6.67 ab

Letters compare Se species among the treatments. Different letters in the same column indicate significant differences in Tukey test results.

Table S2: Correlation coefficients among Se species in soybean grains.

Variables	SeCys	MeSeCys	Selenite	SeMet	Selenate
MeSeCys	0.07^{ns}	-	-	-	-
Selenite	-0.22^{ns}	-0.05^{ns}	-	-	-
SeMet	-0.78^{*}	-0.41^{*}	0.20^{ns}	-	-
Selenate	0.52^{*}	0.37^{*}	-0.28^{ns}	-0.92^{*}	-
Se-total	-0.34^{*}	0.16^{ns}	0.39^{*}	0.34^{*}	-0.36^{*}

^{ns} non-significant correlation; * Pearson’s correlations significant.
